# Feasibility of omega-3 fatty acid supplementation as an adjunct therapy for people with chronic obstructive pulmonary disease: study protocol for a randomized controlled trial

**DOI:** 10.1186/1745-6215-14-107

**Published:** 2013-04-24

**Authors:** Ashley S Fulton, Alison M Hill, Marie T Williams, Peter RC Howe, Peter A Frith, Lisa G Wood, Manohar L Garg, Alison M Coates

**Affiliations:** 1 Nutritional Physiology Research Centre, Sansom Institute for Health Research, School of Health Sciences, University of South Australia, City East Campus, Frome Road, Adelaide, South Australia 5000, Australia; 2 Clinical Nutrition Research Centre, School of Biomedical Sciences & Pharmacy, University of Newcastle, University Drive, Callaghan, New South Wales 2308, Australia; 3 Respiratory Medicine, Flinders University, Faculty of Health Sciences, Repatriation General Hospital, Daws Road, Daw Park, South Australia 5041, Australia; 4 School of Population Health, University of South Australia, City East Campus, Frome Road, Adelaide, South Australia 5000, Australia

## Abstract

**Background:**

There is evidence to support the use of supplementation with long-chain omega-3 polyunsaturated fatty acids (LCn-3PUFA) from oily fish or fish oil for the treatment of various inflammatory diseases such as rheumatoid arthritis. Chronic obstructive pulmonary disease (COPD) is a progressive, terminal disease characterized by persistent airflow limitation, lung and systemic inflammation. To date, one randomized controlled trial has been published that assessed the efficacy of LCn-3PUFA in people with this condition. The aim of this article is to discuss the feasibility of conducting a trial to evaluate fish oil supplementation as adjunct therapy in people with COPD.

**Methods/Design:**

A 16-week parallel, double-blind, randomized, placebo-controlled dietary supplementation trial will be evaluated. Forty participants meeting spirometric and clinical criteria for COPD will be recruited from metropolitan Adelaide, South Australia. Participants will be randomized by minimization, based on a score derived from the modified Medical Research Council Scale for breathlessness, to receive 6 g/day of fish oil (approximately 3.6 g/day of LCn-3PUFA), or placebo (6 g/day of corn oil) capsules. Feasibility outcomes (recruitment, retention, supplement adherence, and time lost to exacerbation) and scientific outcomes (effect size and estimates of variance for inflammatory biomarkers, incorporation of LCn-3PUFA into erythrocytes, small airways function, dyspnea and functional exercise capacity) will be assessed pre- and post-intervention. Key feasibility criteria include recruitment of 40 participants in 52 weeks, 75% participant retention rate, 2% increase in the proportion of long-chain omega-3 fatty acids in erythrocytes, and a positive moderate effect size in at least three efficacy measures.

**Discussion:**

There are a number of challenges in designing supplementation intervention studies with this population. These include the lack of prior data from which to select appropriate primary outcomes or to estimate effect sizes, and the feasibility of continuous supplementation in a population characterized by multiple comorbidities and a high likelihood of exacerbations, potentially requiring hospitalization or change in medication. Upon completion of this protocol, feasibility outcomes will guide the direction of future multicentre dietary interventions in this population.

**Trial registration:**

Australia and New Zealand Clinical Trials Register (ANZCTR): ACTRN12612000158864

## Background

Chronic obstructive pulmonary disease (COPD) is a progressive lung disease characterized by irreversible airway obstruction and systemic inflammation [1]. In 2008, COPD was the fourth leading cause of death worldwide [2], and is predicted to be the third leading cause of death by 2030 [3]. Airway obstruction is caused by inflammation of the bronchi leading to increased mucus secretion (chronic bronchitis) and destruction of lung parenchyma leading to distal airway dynamic compression (emphysema). Air flow is further limited by structural changes that occur as a result of chronic inflammation [1]. The clinical signs and symptoms of COPD include dyspnea, productive cough, wheeze, fatigue, reduced exercise tolerance and repeated respiratory infections. Exacerbations (acute worsening of symptoms leading to a change in medication or management) caused by infection, change of season and air pollution, commonly occur in people with COPD and contribute to the overall severity of the disease [1]. The perception of breathing discomfort known as dyspnea, is one of the most common life impairing symptoms of COPD [4]. Dyspnea restricts and reduces physical activity in people with COPD, which over time leads to a more sedentary lifestyle, reduces cardiorespiratory fitness and quality of life [4].

In people with COPD, both airway and systemic inflammation occur. It is unclear whether systemic inflammation is pathological or results from an overflow of inflammatory mediators from the lung [5]. Nonetheless, the systemic inflammation associated with COPD can impair skeletal muscle function and mass, cardiovascular function, skeletal integrity and mental health, and therefore increase the risk of diseases such as osteoporosis, cardiovascular disease, diabetes and depression [5-8]. Dietary interventions which have been shown to be beneficial in the treatment of inflammatory diseases such as rheumatoid arthritis and cardiovascular disease, such as supplementation with long-chain omega-3 polyunsaturated fatty acids (LCn-3PUFA), might also be beneficial for people with COPD.

Polyunsaturated fatty acids (omega-3 and omega-6) are essential fatty acids that must be consumed in the diet as the human body is unable to synthesize them. They can be further desaturated and chain elongated to form LCn-3PUFA or LCn-6PUFA, respectively. The major LCn-3PUFAs, docosahexaenoic acid (DHA) and eicosapentaenoic acid (EPA), can also be sourced directly from consumption of fish, while the major long-chain omega-6 fatty acid (LCn-6PUFA), arachidonic acid (AA), can also be sourced through the consumption of meat, dairy and eggs [9]. Both LCn-3PUFA and LCn-6PUFA are essential for functions in multiple systems in the body, but are particularly important in the inflammatory response. The metabolism of omega-3 and omega-6 fatty acids have different effects in the body: EPA produces less potent inflammatory eicosanoids than AA; EPA, DHA and AA differ in their roles as mediators of inflammation resolution [10]. Increased consumption of EPA and DHA results in proportional increases of these fatty acids in inflammatory cells at the expense of AA [11].

The production of inflammatory mediators is also influenced by LCn-3PUFA at a cellular level through changes in gene transcription. An important gene transcription factor in the inflammatory response is nuclear factor kappa beta (NF-κB), which must be activated before it can translocate into the nucleus to commence gene transcription [10,12]. It is suggested that DHA and EPA are able to inhibit the activation of NF-κB, thereby reducing the transcription of a number of inflammatory cytokines and chemokines [10].

A preliminary review of the literature was conducted in March 2013. Major databases with a health focus (for example, Medline, Embase, Cochrane Library) and English language clinical trial registries (for example, Clinical Trials.gov) were searched for studies that investigated the intake of polyunsaturated fatty acids (PUFAs) in human adults with COPD. Conference abstracts were excluded. This search identified five published studies and three study protocols (one of which is the proposed study); details of these studies can be found in Table 1. An additional published study was identified although the study has been retracted due to falsified data and was therefore excluded [13].

**Table 1:** Summary of published studies and proposed trials of omega-3 fatty acid supplementation in chronic obstructive pulmonary disease (COPD)

**Author**	**Broekhuizen *et al*.**	**Shahar *et al*.**	**McKeever *et al*.**	**de Batlle *et al*.**	**Hirayama *et al*.**	**Barr (Investigator) protocol**	**Engelen (Investigator) protocol**	**Proposed study**
Year	2005	1994	2008	2011	2010	2012 (recruiting)	2012 (recruiting)	2012
Country	Netherlands	United States	Netherlands	Spain	Japan	United States	United States	Australia
Study type	Randomized controlled trial	Cross sectional	Cross sectional	Cross sectional	Case controlled	Interventional pilot	Randomized controlled trial	Interventional feasibility
Retrospective/prospective	Prospective	Retrospective	Retrospective	Retrospective	Retrospective	Prospective	Prospective	Prospective
Sample size (n)	102	7,902	13,820	250	618	40-45	77	40
n with COPD	102 (100%)	197 (2.5%)	553 (4%)	250 (100%)	278 (45%)	40-45 (100%)	Unclear	40 (100%)
Definition of COPD	PBD, ≥ GOLD stage 2 (at least 50%≤FEV_1_ <80% predicted)	FEV_1_ ≤65%	≥ GOLD stage 2 (at least 50%≤FEV_1_ <80% predicted) Not PBD	PBD FEV_1_/FVC <0.70	PBD FEV_1_/FVC <0.70	PBD FEV_1_/FVC <0.70 and PBDFEV_1_ <65% predicted	PBD FEV_1_ <70% predicted	PBD FEV_1_/FVC <0.70
Mean Age (SD)	A- 64 (10)	54 (6)^e^	42.2 (11.2)	68 (8)	65.8 (6)^e^	N/A	N/A	N/A
	P- 62 (8)							
Mean PBD FEV_1_ % (SD)	A- 38.2 (13.1)	90 (18)^e^	Not reported	53 (16)	Not reported	N/A	N/A	N/A
	P- 35.8 (15.1)							
Intervention	PUFA blend^d^	No	No	No	No	Fish oil	Fish oil	Fish oil
Placebo	Palm oil (80%) & sunflower oil (20%)	No	No	No	No	Corn oil	Olive oil	Corn oil
Control group	Yes	No	No	No	Yes	Yes	Yes	Yes
Supplementation duration	8 weeks	N/A	N/A	N/A	N/A	6 months (2g EPA/ 0.5g DHA per day)	4 weeks (3.5g or 2.0g omega-3 per day)	4 months (3.4g omega-3 per day)
Six-minute walk test	No	No	No	No	No	Yes	No	Yes
Bicycle ergometry	Yes	No	No	No	No	No	No	No
Pulmonary function	Yes	Yes	Yes	Yes	Yes	Yes	No	Yes
Blood biomarkers	Yes	No	No	Yes	No	Yes	Yes	Yes
HADS	No	No	No	No	No	No	No	Yes
Dyspnea questionnaire	No	No	No	No	Yes	No	No	Yes
QOL questionnaire	No	No	No	No	No	Yes	No	Yes
FFQ	No	Yes (61-item, intake past year)	Yes (178 item, past years intake)	Yes (122 item, past 2 years intake)	Yes (138 item, past 5 years intake)	No	No	Yes (74 item, past years intake)
Endothelial function	No	No	No	No	No	Yes	No	No
Muscle strength(skeletal & respiratory)	Yes	No	No	No	No	No	Yes	No
Muscle protein synthesis & breakdown	No	No	No	No	No	No	Yes	No
Oxidative capacity	No	No	No	No	No	No	Yes	No
Duel-Energy X-ray absorptiometry	No	No	No	No	No	No	Yes	No
Statistical test	Linear regression	Logistic regression	Nonlinear regression	Logistic regression	Logistic regression	Association between n-3 intake and endothelial function	Association between n-3 intake and protein synthesis	ANOVA
	Comparison PUFA and placebo group after 8 week intervention.	Association between n-3 intake and COPD	Association between n-6 intake and COPD	Association between PUFA and biomarkers	Association between PUFA intake and COPD			
Results	Greater ↑ exercise capacity in PUFA compared to placebo group*.	COPD strongly and inversely associated with intake of n-3 fatty acids*^b^	No association between COPD and n-3 intake.	↑ ALA associated with↓ TNF-α *^a^	↑ prevalence COPD associated with, ↓ PUFA intake *^b^, ↓ n-6 intake *^b^, ↓ n-3 intake *^b^	N/A	N/A	N/A
			↑ intake of four of the n-6 fatty acids associated with ↑ risk of COPD*^c^.	↑ LA and AA associated with ↑ CRP *^c^				
	PUFA no effect on FEV_1_ or muscle strength.							
	No change in blood biomarkers in either group.			↑ AA associated with ↑ IL-6 *^c^				

A, active; ALA, alpha linoleic acid; ANOVA, analysis of variance; AA, arachidonic acid; CRP, C-reactive protein; DHA, Docosahexaeneoic acid; EPA, eicosapentaenoic acid; FEV1, forced expiratory volume in 1 second; FFQ, food frequency questionnaire; GOLD, Global initiative for chronic obstructive lung disease; HADS, Hospital anxiety and depression scale; IL-6, interleukin-6; LA, linoleic acid; n-3, omega-3 fatty acid; P, placebo; PBD, Post bronchodilator; PUFA, Polyunsaturated fatty acid; QOL, quality of life; 6MWD, six-minute walk distance; TNF-α, tumor necrosis factor-alpha.

*Statistically significant *P* <0.05 ^a^ odds ratio <1, ^b^odds ratio ≥1.0, ^c^odds ratio ≥1.5.

PUFA refers to both omega-3 and omega-6 fatty acid intake.

^d^ PUFA blend consists of 400 mg stearidonic acid, 760 mg gamma-linolenic acid, 1,200 mg alpha-linolenic acid, 700 mg eicosapentaenoic acid and 340 mg docosahexaenoic acid.

^e^Weighted mean.

Broekhuizen *et al*. [14] investigated the effects of PUFAs on the outcome of pulmonary rehabilitation (exercise capacity and reversal of muscle wasting). This study compared the effects of eight weeks of pulmonary rehabilitation combined with a total daily PUFA dose of 3.4 g/day (400 mg stearidonic acid, 760 mg gamma-linolenic acid, 1,200 mg alpha-linolenic acid (ALA), 700 mg EPA and 340 mg DHA) versus a placebo (palm and sunflower oils) in 102 people with COPD. All other included published studies [15-18] investigated associations between omega-3 intake and COPD prevalence or inflammation in different populations.

Overall the results of the five studies varied. Broekhuizen *et al*. [14] reported an increase in maximum load (incremental maximal test) and an increase in duration (fixed load submaximal) bicycle ergometry tests for both groups; however the increase in the PUFA group was significantly greater than the placebo group. PUFA supplementation had no effect on muscle strength or FEV_1_ and neither PUFA supplementation nor pulmonary rehabilitation affected biomarkers of inflammation [14]. McKeever *et al*. [17] reported no significant association between LCn-3PUFA and COPD. Both Shahar *et al*. [18] and Hirayama *et al*. [16] reported inverse associations between LCn-3PUFA intake and the prevalence of COPD, while de Batlle *et al*. [15] reported negative associations between LCn-3PUFA intake and concentrations of inflammatory markers. In studies that reported an association between intake of omega-3 or omega-6 fatty acids and COPD, the strength of the associations was predominantly small (odds ratio 1.5 to 3.5 [19]).

Excluding the current protocol, the search of the clinical trials registries identified two randomized controlled trials currently underway (University of Columbia [20] and Texas A&M University [21]). The University of Columbia study is a pilot randomized controlled trial with a lower dose of LCn-3PUFA (2.5 g/day DHA + EPA) than the proposed study, but for a longer supplementation period. The Texas A&M University randomized controlled trial has a similar dose to the proposed study (3.5 g/day DHA + EPA) but for a shorter supplementation period (8 weeks). There are similarities between the three studies (for example, outcomes and omega-3 dose) although the primary focus of the three proposed studies differs, with Columbia University focusing on endothelial function, Texas A&M focusing on muscle repair and damage, while the proposed study focuses on feasibility for a larger RCT.

In theory, LCn-3PUFA from fish oil may improve efficacy of current treatments for COPD by reducing inflammation. Given the paucity of data for fish oil supplementation trials in people with COPD, there are currently no data to guide study design decisions such as appropriate outcome measures (including the primary outcome), likely effect sizes or sample size determination. COPD is a complex condition where comorbidities and exacerbations are to be expected, both of which may confound the effect of, or adherence to supplementation. In addition, slow recruitment and high dropout rates have been reported in intervention trials for people with COPD [22-24]. The driver for this proposed research is the hypothesis that LCn-3PUFA supplementation as an adjunct therapy will improve health outcomes in people with COPD. However before this hypothesis can be tested, it is important to establish the feasibility of the study protocol and justification for continuing to a larger study.

While the terms ‘pilot study’ and ‘feasibility study’ are often used interchangeably in the literature, Arain *et al*. [25] explains the distinction between the two. A feasibility study seeks to determine if the study is possible, with the available resources and in the current circumstances. A pilot study is used to determine the efficiency of the study protocol as a whole. Thabane *et al*. [26] and van Teijlingen *et al*. [27] recommend undertaking a feasibility study in order to determine recruitment rates, estimate variability of outcomes to assist with sample size calculations or to obtain data to support a funding application.

Given the lack of pre-existing data on which to inform primary outcome and sample size estimates and the known complexities of undertaking intervention studies in people with COPD, this study aims to determine the feasibility of undertaking a randomized controlled study of daily LCn-3PUFA supplementation in people with COPD. The objectives of this study are to determine feasibility within two domains including process (for example, recruitment and retention rates) and scientific (for example, estimate of effect size and variance in outcomes).

## Methods/Design

### Study design

**Figure 1:**
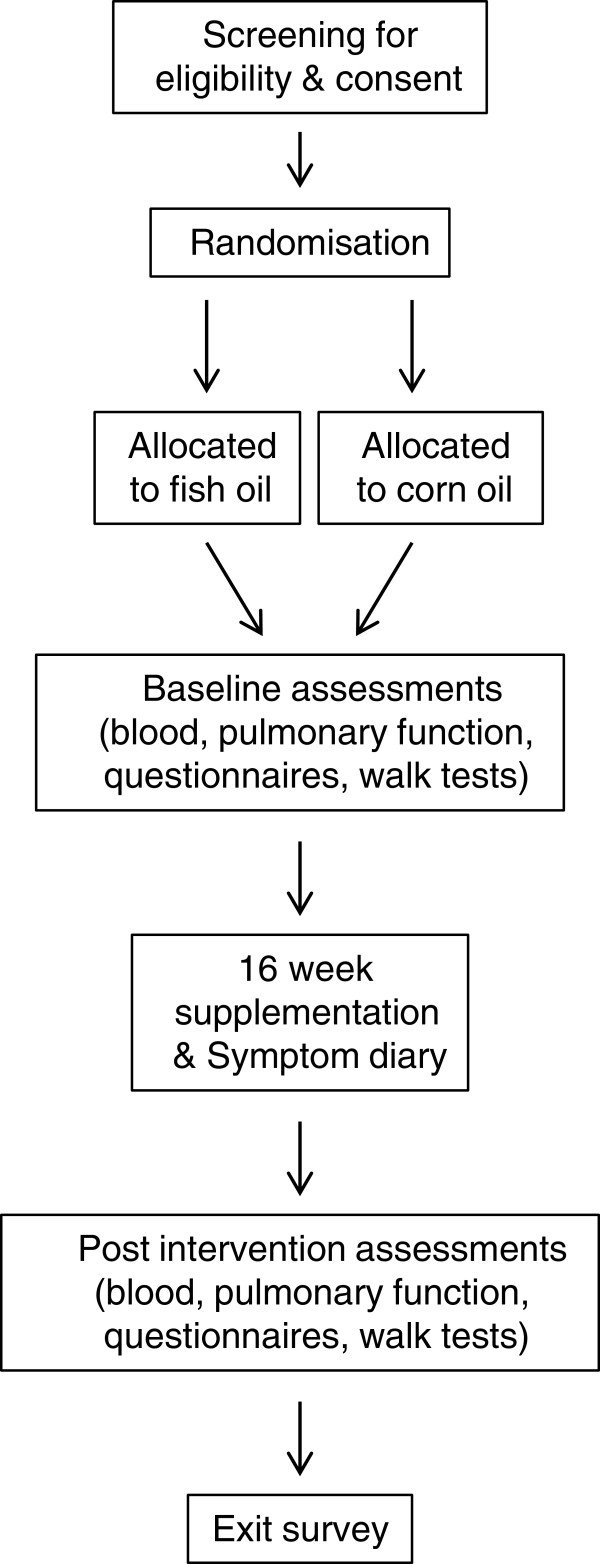
Study overview.

A 16-week parallel, double-blind, randomized, placebo-controlled dietary supplementation trial will be conducted in the Respiratory Medicine department at the Repatriation General Hospital (RGH), Adelaide, South Australia in collaboration with the Nutritional Physiology Research Centre (NPRC), University of South Australia, Adelaide, South Australia and the Clinical Nutrition Research Centre, University of Newcastle, Callaghan, NSW. Participants will attend the RGH on two occasions (pre- and post-intervention) for a series of tests including pulmonary function, venepuncture, well-being questionnaires and functional exercise capacity (see Figure 1).

### Participants

Participants will be recruited from the respiratory trials database (which contains >500 people who have previously indicated their willingness to be contacted for future studies) at the RGH. To be eligible for inclusion, participants are required to be adults (≥18 years of age), have a clinical and spirometric diagnosis of COPD (FEV_1_/FVC <0.7) based on GOLD criteria [1], and stable medication for 28 days prior to screening. Participants will be excluded from the study if they habitually consume LCn-3PUFA supplements, have unstable or very severe COPD (FEV_1_ <30% predicted) [1], have a diagnosis of α-antitrypsin deficiency, are a current smoker, are cachexic (unintentional weight loss of >5% within 12 months [28] or have a body mass index (BMI) ≤18.5 kg/m^2^), are morbidly obese (BMI ≥40 kg/m^2^), are experiencing unstable comorbidities (for example, right heart failure, sleep disorders), have used systemic steroids or antibiotic medication within 28 days prior to enrolment, are diagnosed with respiratory conditions not related to airflow limitation (for example, pulmonary fibrosis), have unstable medication use (change in medication or use in the 6 months prior to enrolment), have participated in a comprehensive pulmonary rehabilitation program in the previous 2 years or are intending to undertake this type of rehabilitation program in the 6 months following study enrolment. Written informed consent will be obtained from all participants.

### Ethics and Trial registration

Ethics approval for this study was granted by the Human Research Ethics Committee of the University of South Australia (project number: 0000026302) and Southern Adelaide Clinical Human Research Ethics Committee (project number: 208.12). The trial was registered on the Australia and New Zealand Clinical Trials registry (ANZCTR) on the 3rd of February 2012 (ANZCTR Number: 12612000158864).

### Sample Size

As this is a feasibility study designed to determine pragmatic aspects of methodology, formal sample size calculations are not appropriate [26,29]. The target sample size for the feasibility study is 40 participants. The target sample is to allow sufficient precision to enable sample size calculations for subsequent studies. Browne [30] suggests that a minimum of 30 participants is required to achieve sufficient precision, a recommendation supported by Lancaster *et al*. [31]. The target sample of 40 enrolled participants will allow completion of 30 subjects, allowing for a 25% participant dropout rate. A key feasibility criterion is the ability to recruit a target sample of 40 participants within 52 weeks.

### Randomization

Subjects will be randomly allocated by minimization [32,33], based on modified Medical Research Council (mMRC) scale for breathlessness scores, to either the fish oil group or the placebo group. The prognostic factor that will be considered in the minimization model will be mMRC score (0, 1, 2, 3 or 4).

### Dietary intervention

Participants will be required to take six 1 g capsules orally per day for 16 weeks. The fish oil group will consume capsules containing EPAX 6000 TG/N omega-3 concentrate (EPAX, Oslo, Norway). Each capsule contains 600 mg of omega-3 fatty acids, which is specified to contain 200 mg of DHA and 300 mg of EPA. It should be noted that while the recommended upper limit of LCn-3PUFA is 3 g/day [34], a number of studies have used higher doses (3.4 g/day to 6.6 g/day) without any reported side effects [35,36]. The placebo group will receive capsules that are identical in appearance containing corn oil (predominantly comprised of linoleic, oleic and palmitic acids) (EPAX, Oslo, Norway). All capsules are flavored to mask the taste and odor. Participants will continue their usual medical treatment throughout the supplementation period. Self-reported medication and supplement use will be collected pre- and post-intervention.

### Blinding

Capsules will be prepackaged into opaque containers and labeled as A or B with the investigator details and supplementation instructions. A staff member of the NPRC external to the project will determine the computer-generated randomization schedule. Capsules will be securely stored and will be dispensed in opaque containers by the clinical trials pharmacist at the RGH, thus ensuring blinding of both the participants and investigators.

### Anthropometry

Height will be measured to the nearest 0.5 cm using a wall-mounted stadiometer (Seca 240, Seca, Hamburg, Germany) and mass will be measured to nearest 0.1 kg using a calibrated digital precision scale (Wedderburn DS-520, Weddernburn, Australia) at both assessment visits. BMI will be calculated from these data as mass (kg) /height (m)^2^.

### Dietary intake

Total energy, macronutrient consumption, and micronutrient consumption will be estimated at baseline using a 74-item food frequency questionnaire from the Cancer Council of Victoria, Australia [37]. The food frequency questionnaire has been validated [38] for use in human dietary intervention trials. The food frequency questionnaire will be repeated post-intervention to determine whether there were any changes in diet throughout the supplementation period.

### Comorbid status

Comorbid status will be determined using two questionnaires: the Charlson index [39], which weights comorbid diseases based on their association with mortality, and the Rx-Risk comorbidity index [40], which uses drug classifications to determine comorbid diseases. Both have been included because there is no consensus on the best method for quantifying comorbid status; therefore, the suitability of both indices will be assessed.

### Feasibility outcomes and criteria

Within the four domains of feasibility (process, resources, management and scientific) there are specified outcomes in the process and resource domains that will be assessed throughout the study against feasibility criteria (see Table 2).

**Table 2:** Feasibility outcomes and key feasibility criteria

**Outcome**	**Outcome measure**	**Key feasibility criteria**
*Process*		
Recruitment rate	Number of participants recruited per week.	Recruit 40 participants in 52 weeks.
Retention rate	Number of participants completing the intervention.	Complete the intervention in 80% of all recruited participants.
Supplement adherence rate	Symptom diary.	
	Change in RBC total LCn-3PUFA content.	≥2% change in total RBC LCn-3PUFA.
	Capsule count at study completion.	≥80% of capsules consumed.
Refusal rate	Number of identified volunteers who decline to participate/randomize.	
Time lost to exacerbation	Symptom diary.	
*Scientific*		
Supplement safety	Reported symptoms from supplement.	
Estimate effect size	Listed scientific outcomes.	A positive moderate effect size (≥0.2) in at least CRP, dyspnea and FEF_25-75_.
Estimate variance	Standard deviations of listed outcomes.	

CRP, C-reactive protein; FEF_25-75_, forced expiratory flow between 25 and 75% of forced vital capacity; LCn-3PUFA, long chain omega-3 polyunsaturated fatty acid; RBC, red blood cell.

#### Process outcome measures

A number of process outcomes will be assessed throughout the study including participant recruitment and retention, supplement adherence, time lost to exacerbation and participant experiences and perspectives about participating in the study. Participant recruitment and retention will be assessed descriptively by recruitment and refusal rates, while retention will be determined by the number of enrolled participants who complete the study protocol.

Supplement adherence will be assessed in three ways. Firstly, by determining changes from baseline to post-intervention in the LCn-3PUFA content of erythrocytes, as the LCn-3PUFAs are incorporated into erythrocyte membranes in a dose-dependent and time-dependent manner [41]. Secondly, a symptom diary will be used to document self-reported supplementation consumption including whether the capsules were consumed each day, how many capsules were consumed and whether any side effects were experienced such as eructation or heartburn. Thirdly, participants will be asked to return all unused capsules at the completion of the study and the remaining capsules will be counted.

The time lost to exacerbation (change in usual symptoms potentially leading to a change in medication, change in usual activities or unscheduled visit to a doctor) will be determined using a symptom diary in the format of one month per page. The diary enables the assessment of exacerbation frequency and severity during the supplementation period. The number of exacerbation days will be counted from the diary, while an exacerbation severity score will be calculated using methods adapted from those described by Effing *et al*. [42]. Data collected from the symptom diary will be used to determine the frequency of exacerbation, whether periods of exacerbation impact upon supplement consumption and whether periods of exacerbation have a confounding effect on supplementation or study outcomes. This information will be used to determine whether exacerbations during the supplementation period should be an exclusion criterion in a subsequent study.

Participant experience and perspectives will be gathered using a structured telephone interview on exit from the study. The structured interview will have four main sections including: 1) the assessments (for example, opinions about the test battery, use of the symptom diary); 2) the supplement (for example, adherence, symptoms and which arm of the trial they believed they were on, any adverse effects experienced); 3) the trial visits and the trial itself (for example, length of the visits, best and worst things about participating in the trial, participation in future trials); and 4) the participants (for example, behavioral changes during trial and life events external to the trial which could impact on trial outcomes).

### Scientific outcome measures

**Figure 2:**
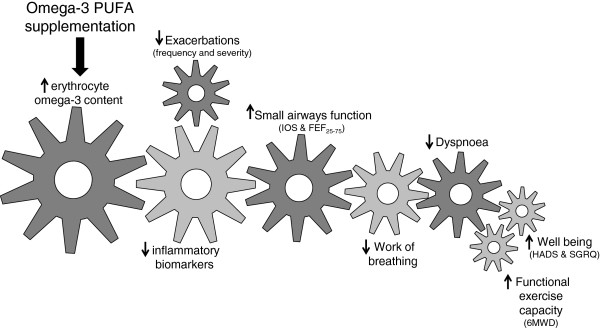
**The proposed relationship between omega-3 fatty acid supplementation mechanisms and outcomes of interest.** 6MWD, six minute walk distance; CRQ, chronic respiratory questionnaire; FEF_25-75_, forced expiratory flow between 25 and 75% of forced vital capacity; HADS, hospital anxiety and depression scale; IOS, impulse oscillometry; PUFA, polyunsaturated fatty acid.

A number of scientific outcomes have been included in order to determine which are the most appropriate for use in a subsequent study. Scientific outcomes have been selected which reflect stages in the theoretical mechanism cascade from cellular (for example, inflammatory biomarkers) to patient-centered (dyspnea, exercise capacity, quality of life) outcomes. Figure 2 presents the proposed cascade between LCn-3PUFA supplementation and the outcome measures.

#### Blood biomarkers

Venous blood samples will be obtained after a 10 to 12-hour overnight fast by venepuncture (EDTA-containing tubes) and will be centrifuged within two hours of sampling. Plasma will be removed and erythrocytes will be washed with saline. Aliquots of plasma and erythrocytes will be stored at −80°C until analysis. Relative proportions of individual fatty acids in erythrocyte phospholipids will be assessed using a method adapted from previously established methods [43-45] and described in detail by Milte *et al*. [46]. In this process fatty acids will be extracted and transmethylated and the methyl esters will be separated and quantified using a Shimadzu 2010 gas chromatograph (Shimadzu, Milan, Italy). Fatty acid methyl esters will be identified by comparing retention times to those of authentic lipid standards (GLC-463, Nu-Chek Prep Inc. Elysian, MN, USA). Inflammatory biomarkers including tumor necrosis factor (TNF-α), interleukin-1 beta (IL-1β) and interleukin-6 (IL-6) and the anti-inflammatory biomarker interleukin-10 (IL-10) will be analyzed in duplicate using multiplex Luminex analyzer (Luminex B.V., Oosterhout, The Netherlands) assays. Intra- and inter-assay coefficients of variation (CV) are 5 to 14% and 4 to 18%, respectively. High sensitivity C-reactive protein will be measured using an automated spectrophotometric analyzer (Konelab, Model 20xTi; Thermo Electron, Waltham, MA, USA); the CV for this determination is 2.3%.

#### Pulmonary function

Lung function tests will follow standardized American Thoracic Society/ European Respiratory Society taskforce guidelines [47] to determine forced expiratory volume in 1 second (FEV_1_), forced vital capacity (FVC), inspiratory capacity (IC) and vital capacity (VC), which enables the diagnosis and severity classification of COPD. Small airways function will be assessed using two methods: forced expiratory flow will be measured at various time points (FEF_25-75_, FEF_25_, FEF_50_, FEF_75_) [47] and impulse oscillometry will be used to determine respiratory impedance through the application of small pressure oscillations at the mouth during normal breathing [48]. Single-breath determination of carbon monoxide (CO) uptake will be used to determine the gas transfer efficiency of the lung tissue as the diffusing capacity of carbon monoxide (*D*L, CO) [49], which is reduced in the presence of emphysema and some other conditions. Lung volumes will be determined using body plethysmography [50].

#### Dyspnea

The sensation of dyspnea is currently considered to have three domains (sensory quality, intensity and unpleasantness). The Dyspnoea-12 questionnaire will be used to assess the sensory quality of breathlessness. Visual analogue scales (10 cm horizontal line anchored on the left as unaffected) will be used to quantify unpleasantness (not unpleasant to worst unpleasantness imaginable) and intensity (no breathlessness to the worst possible breathlessness) of dyspnea. Assessments of dyspnea will be conducted at rest (average sensation over the past two weeks) and immediately after the six-minute walk test (breathlessness induced by exercise). The mMRC score will be used to assess dyspnea related functional impairment, with a scoring system of 0 to 4 (where 4 represents severe breathlessness related impairment). The mMRC scale is widely used in people with COPD and is a reliable (intraclass correlation = 0.82) [51] tool with acceptable concurrent validity (*r* = 0.70) [52].

#### Functional exercise capacity

The six-minute walk test will be used to determine change in functional exercise capacity (six-minute walk distance). Participants walk on a hard flat surface as far as they can in six minutes (one lap up and back = 40m), stopping for breaks if required. The test will be conducted twice with the maximum walk distance used for analysis. The six-minute walk test is a reliable (CV = 8%, intra-class correlation coefficient = 0.93) measure when conducted under standardized conditions [53,54] and is commonly used as an indicator of functional exercise capacity in the COPD population [55-57] as well as other chronic diseases such as cardiovascular disease [58].

#### Well-being

The Hospital Anxiety and Depression Scale (HADS), a widely used reliable validated tool [59] will be used to assess symptoms of depression and anxiety [60]. The HADS has been used previously in the COPD population with good reliability (Cronbach’s α = 0.86 (part A) and 0.87 (part B)) [61]. The chronic respiratory questionnaire (CRQ) is a self-administered respiratory specific quality of life questionnaire with good reliability [62] and validity [63]. The questionnaire assesses four domains including dyspnea, fatigue, emotional function and mastery [62].

### Data analysis

SPSS version 17 for WINDOWS [International Business Machines Corporation (IBM), New York, USA] will be used for analyses. Data will be cleaned, checking for errors or missing values. Continuous data will be checked for normality of distribution and non-normally distributed data will be transformed where possible. Scatter plots will be used to determine nature/shape of relationships before applying linear regression.

The primary focus of this study is feasibility, which will be analyzed using descriptive statistics and frequency tables. The key feasibility outcomes are:

1. Recruit 40 participants in 52 weeks;

2. Complete the intervention in 75% of all recruited participants;

3. ≥2% change in total erythrocyte LCn-3PUFA and ≥80% of capsules consumed (estimated from remaining capsules);

4. A positive moderate effect size (≥0.2) in at least three scientific outcome measures (between active and placebo groups).

Analysis of variance (ANOVA) with repeated measures will be used to estimate the effect size of LCn-3PUFA versus placebo supplementation on the scientific outcome measures (six-minute walk, pulmonary function tests, questionnaire scores, blood biomarkers of inflammation and symptoms (for example, high sensitivity C-reactive protein, dyspnea and impulse oscillometry)). Progressive Bonferroni corrections will be used to correct for the effects of multiple outcomes. Comorbid status and severity of exacerbations will be assessed as potential confounders using multiple regression analysis.

### Assessment administration

All measures will be completed in the respiratory medicine laboratory at the RGH, and will be administered by the same investigator (AF, excluding lung volumes and gas transfer), which will be conducted by respiratory function scientists). The testing sessions will last approximately two hours. Tests will be conducted in the following order: blood collection, pre-bronchodilator spirometry, gas transfer (administration of bronchodilator prior to), lung volume, first six-minute walk, questionnaires, post bronchodilator spirometry and the second walk test. All assessments will be timed and compared to the estimated completion time as a feasibility outcome. All blood analysis including preparation (saline wash) will be conducted by the same investigator (AF, excluding Luminex analysis, for which AF will assist a NPRC staff member). Initial preparation of samples will occur at the SA Pathology laboratory at the RGH, and all analyses (erythrocyte fatty acid extraction and biomarker analysis) will occur in the NPRC laboratory (AF and other NPRC laboratory staff).

## Discussion

People with COPD have an accumulation of inflammatory cells in the respiratory tissue that causes damage to the lungs [64]. This is further compounded by the effects of systemic inflammation such as reduced muscle function and skeletal integrity, which are often associated with comorbidities such as cardiovascular disease and osteoporosis [5-7]. The anti-inflammatory properties of LCn-3PUFA have been beneficial in other inflammatory diseases with recent systematic reviews in rheumatoid arthritis and cardiovascular disease concluding that the benefits of LCn-3PUFA are modest but clinically relevant [65,66]. In people with rheumatoid arthritis all included studies showed some clinical benefit with LCn-3PUFA supplementation [66], while in cardiovascular disease those supplemented with LCn-3PUFA had a 10% reduction in the number of cardiovascular events [65]. Given the impact of lung and systemic inflammation, dietary supplementation with LCn-3PUFA may be beneficial for people with COPD.

The current evidence for benefits of LCn-3PUFA in people with COPD is limited; there has been one RCT, although not exclusively using LCn-3PUFA. The majority of studies focus on the association between self-reported intakes of LCn-3PUFA and prevalence of COPD (Table 1). Most of these studies used spirometry to diagnose COPD; however, the definition of COPD (Global initiative for chronic obstructive pulmonary disease (GOLD) stage [1]) or spirometric criterion varied between studies. Shahar *et al*. [18] and McKeever *et al*. [17] did not use post-bronchodilator spirometry in the diagnosis of COPD, and therefore irreversible airflow limitation (a requirement of COPD) could not be confirmed.

The RCT conducted by Broekhuizen *et al*. [14] did not test effects of LCn-3PUFA specifically; it was combined with other PUFAs. Hence it is unclear whether LCn-3PUFA alone was responsible for the observed changes in outcomes or whether these changes were attributable to the combinations of nutrients. The dose of LCn-3PUFA (EPA and DHA) used in the Brekhuizen *et al*. [14] study was approximately 1 g/day for eight weeks. It is suggested by Calder [67] that a minimum dose of 2 g/day is required to elicit the anti-inflammatory properties of omega-3 fatty acids (DHA + EPA). This study combined supplementation with pulmonary rehabilitation, the beneficial effects of which are well documented in people with COPD [68]. Broekhuizen *et al*. [14] suggests that the increases in exercise capacity could be a result of increased fatty acid availability and oxidative capacity which could be related to changes in muscle fiber type through exercise. Pulmonary rehabilitation may have facilitated the reported effects of PUFAs in COPD; however the effects of PUFA supplementation alone on health outcomes in people with COPD are still unknown and warrant investigation.

The proposed study aims to determine not only the feasibility of the study protocol but also which outcomes might be appropriate to include in a larger trial. In order to achieve these aims, a number of outcomes have been included, ranging from cellular, such as blood biomarkers of inflammation, to patient centered measures, such as dyspnea and exercise capacity. While there are likely to be changes in cellular outcomes such as inflammatory biomarkers, it is possible that a corresponding change in clinically relevant outcomes such as dyspnea may not be achieved. This is likely to influence uptake of LCn-3PUFA supplementation by people with COPD and health care practitioners as a worthwhile addition to current best practice.

Studies in fish oil supplementation have previously shown good adherence with the supplementation protocol [41]. Adherence in people with COPD is likely to be similar to that shown in previous studies; however, exacerbations are common in this population and depending on the severity, may lead to changes in medication and hospitalization during the supplementation period. During these periods of time supplementation adherence may be affected. A number of other factors may also influence adherence, such as comorbidities (notably anxiety and depression) and multiple medications associated with the COPD and/or the comorbidities. The dose and duration of supplementation in the current protocol is similar to that used in other studies of fish oil and chronic inflammatory conditions (for example, rheumatoid arthritis). However, as suggested above, it is possible that COPD patients may find it challenging to consume their usual medications plus fish oil capsules. By providing concentrated capsules containing more (600 mg/g) of the active fatty acids and lesser of unnecessary fat, we expect improved compliance as this reduces the number of capsules required to achieve the desired dose.

Pilot and feasibility studies are often disregarded and overlooked within the literature but have the potential to provide valuable information [27]. For example, at the completion of a pilot or feasibility study, reporting the ‘challenges faced’ has the potential to assist other researchers in trial design [27]. Crucially the pilot or feasibility trial will provide information about a population that is not currently available such as ease (or difficulty) in recruitment, and it may also highlight issues with the study protocol not evident during the planning phase. These issues could be crucial to the success or failure of a large study.

In summary, while LCn-3PUFA supplementation in people with COPD may be beneficial there are a number of unknown factors such as supplement adherence, appropriate sample size and choice of outcome measures. The aim of this study is to determine the feasibility of the study and to provide data to guide the design of future studies in dietary supplementation studies in people with COPD.

### Trial status

Not yet recruiting.

## Abbreviations

AA: Arachidonic acid; ALA: Alpha linoleic acid; ANZCTR: Australia and New Zealand Clinical Trials Registry; BMI: Body mass index; COPD: Chronic obstructive pulmonary disease; CO: Carbon monoxide; CRP: C-reactive protein; CRQ: Chronic respiratory questionnaire; CV: Coefficient of variation; DHA: Docosahexaenoic acid; DL,CO: Diffusing capacity of the lung to carbon monoxide; EPA: Eicosapentaenoic acid; FEF: Forced expiratory flow; FEV1: Forced expiratory volume in 1 second; FFQ: Food frequency questionnaire; FVC: Forced vital capacity; GOLD: Global initiative for chronic obstructive lung disease; HADS: Hospital anxiety and depression scale; IC: Inspiratory capacity; IL-1β: Interleukin 1 beta; IL-10: Interleukin 10; LA: Linoleic acid; LCn-3PUFA: Long chain omega-3 polyunsaturated fatty acid; LCn-6PUFA: Long chain omega-6 polyunsaturated fatty acid; mMRC: modified Medical Research Council; NF-κB: Nuclear factor kappa beta; NPRC: Nutritional Physiology Research Centre; PBD: Post bronchodilator; PUFA: Polyunsaturated fatty acid; QOL: Quality of life; RCT: Randomized controlled trial; RGH: Repatriation General Hospital; TNF-α: Tumour necrosis factor alpha; VC: Vital capacity; 6MWD: 6-minute walk distance.

## Competing interests

The authors declare that they have no competing interests.

## Authors' contributions

AC, AH and MW conceived the idea. AH, MW, PH, AC, PF, LW, MG and AF contributed to the development of the protocol. AF, MW, PH, AC and AH drafted the manuscript. PF, LW, MG provided critical revision of the manuscript. All authors read and approved the final manuscript.
